# Stramonium in Neuralgia

**Published:** 1861-05

**Authors:** 


STRAMONIUM IN NEURALGIA.—Dr. A. Young, in a communication to the *Chicago Medical Examiner*, says, that in his experience stramonium is an unfailing remedy in neuralgia. He prescribes one grain of Tilden's extract of stramonium leaves every two or three hours until the system is decidedly affected, as indicated by dilated pupil, disordered vision, vertigo, etc. It should, he thinks, always be given to this extent, and will seldom need repetition.—*Med. and Surg. Reporter.*

